# A rabbit model for experimental alveolar cleft grafting

**DOI:** 10.1186/s12967-017-1155-2

**Published:** 2017-02-24

**Authors:** Mohammad Kamal, Lars Andersson, Rene Tolba, Alexander Bartella, Felix Gremse, Frank Hölzle, Peter Kessler, Bernd Lethaus

**Affiliations:** 1 Department of Cranio-Maxillofacial Surgery, Maastricht University Medical Center, P. Debyelaan, Postbus 5800, 6202 AZ Maastricht, The Netherlands; 2 Department of Oral and Maxillofacial Surgery, RWTH Aachen University, Pauwelsstraße 30, 52074 Aachen, Germany; 3 Department of Surgical Sciences, Faculty of Dentistry, Health Sciences Center, Kuwait University, 13110 Safat, Kuwait; 4 Institute for Laboratory Animal Science and Experimental Surgery, RWTH Aachen University, Pauwelsstraße 30, 52074 Aachen, Germany; 5 Department of Experimental Molecular Imaging, RWTH Aachen University, Pauwelsstraße 30, 52074 Aachen, Germany

**Keywords:** Animal testing, Cleft lip and palate, Grafting, Tissue-engineering, Rabbit

## Abstract

**Objectives:**

The purpose of the present study was to develop an animal model for creating alveolar cleft defects with properly simulated clinical defect environment for tissue-engineered bone-substitute materials testing without compromising the health of the animal. Cleft creation surgery was aimed at creating a complete alveolar cleft with a wide bone defect with an epithelial lining (oral mucosa) overlying the cleft defect.

**Methods:**

A postmortem skull of a New Zealand White (NZW) rabbit skull (*Oryctolagus cuniculus*) underwent an osteological and imaging survey. A pilot postmortem surgery was conducted to confirm the feasability of a surgical procedure and the defect was also radiologically confirmed and illustrated with micro-computed tomography. Then, a surgical in vivo model was tested and evaluated in 16 (n = 16) 8-week-old NZW rabbits to create in vivo alveolar cleft creation surgery.

**Results:**

Clinical examination and imaging analysis 8 weeks after cleft creation surgery revealed the establishment of a wide skeletal defect extending to the nasal mucosa simulating alveolar clefts in all of the rabbits.

**Conclusions:**

Our surgical technique was successful in creating a sizable and predictable model for bone grafting material testing. The model allows for simulating the cleft site environment and can be used to evaluate various bone grafting materials in regard to efficacy of osteogenesis and healing potential without compromising the health of the animal.

## Background

Congenital alveolar cleft is a malformation occurring as a result of non-fusion of primary palate during weeks 4–12 of gestation. The goal of alveolar cleft repair is to establish bony continuity of the alveolar ridge in the maxilla, seal the communication oro-nasal communication, and create a favorable anatomy for dental rehabilitation [1–3]. Reconstruction of the these defects is done via the alveolar cleft bone grafting procedure using autologous bone, allogenic and xenogeneic bone grafting materials, along with various tissue-engineered bone replacement materials [4–8].

Optimising the quality of the existing bone grafting materials and looking for novel and better bone-substitute materials is crucial in improving the clinical outcome. Experimental testing of various grafting materials requires the pre-establishment of a proper biological model to conduct experimental studies and evaluate the clinical effect with respect to osteogenesis and healing. Animal models with simulated alveolar clefts are considered appropriate as an experimental model for testing of clinical interventions. Several animal models have been utilized for testing of alveolar cleft grafting materials including mice, rats, rabbits, cats, dogs, swines, goats, sheep and monkeys [4, 9–24].

Development of alveolar clefts in experimental animals can be achieved, either surgically created or congenitally induced in utero during embryonic development [19, 25–27]. Previous studies on in utero congenitally induced models reported increased need of technical expertise, concurrent multiple fetal malformations, and an increased incidence of intrauterine fetal death and abortions. In addition, several studies reported that newborn animal models with lip defects were less cared by their mothers and some being subjected to cannibalism [28–30]. Next to congenitally induced models, surgically created alveolar clefts in animals also seem suitable to experimental studies regarding histologic and biomechanical properties of bone grafting material.

Moreover, it is essential to allow proper timing for healing of the defect and establish an alveolar cleft of appropriate width mimicking the human scenario of a skeletal defect extending to the nasal mucosa and the adjacent teeth and be suitable for clinical testing. Some earlier reported cleft models do not correspond to the clinical situation since a bone defect is created and filled in the same session. This is not in accordance to the real situation in which the defect is covered by epithelial lining. Hence, it is important to achieve a bony cleft with its surfaces covered with healthy mucosal tissue at the time of placement of graft. For this reason it is necessary to first create the bone defect and then in a second stage surgery, after healing with mucosal lining of the cleft has been achieved, place the grafting material, otherwise the defect is not corresponding to the real clinical situation. Moreover, compared with congenitally induced alveolar clefts, surgically created cleft models in animals can easily be created and allow for controlling the size and extent of the bony cleft and properly position the overlying soft tissue to serve the purpose of the model.

Animals used in biomaterial bone research include small animals, such as mice, rats, guinea pigs, and rabbits, and large animal category mostly goats, dogs, and primates [31–33]. Rodent models have inherent limitations when compared to larger models, including rabbits. Rodents have smaller long-bones, more fragile cortex, and do not show Haversian-type remodeling in the cortex [31]. Rabbits are considered the largest animals in the small animals category, and hence less susceptible to elaborate and exhaustive additional clearance requirements usually implemented by the central ethical committees. They are non-aggressive, easy to observe, have quicker vital capacity in terms of gestation and maturity, and can be locally bread [31–33]. The histology of bone in rabbits is not quite similar to bone in humans, and composes of dense Haversian bone and layers primarily vascular longitudinal canals [31–33]. However, similarities in bone mineral density and fracture toughness between rabbits and human have been reported in the literature [32–34]. An essential issue with rabbits that they express rapid skeletal metabolism and increased bone turnover rate, mostly cortical remodeling when compared to primates and some rodents [31–33]. To properly simulate human in vivo environment, the rabbit model is an appropriate animal model for alveolar cleft experimental studies because the rabbit is reproducible, accurate, easy to house and handle, relatively easily anaesthetised, provides large enough area for testing and properly sized mammalian that can bear the trauma of surgery [32, 33].

The aim of this study was to develop a model in New Zealand White (NZW) rabbits, *Oryctolagus cuniculus*, to enable surgically creating of a healed skeletal alveolar defect extending to the nasal mucosa and the adjacent tooth structure as seen in human patients.

## Methods

### Osteological survey of New Zealand White rabbit skull (*O. cuniculus*)

**Fig. 1:**
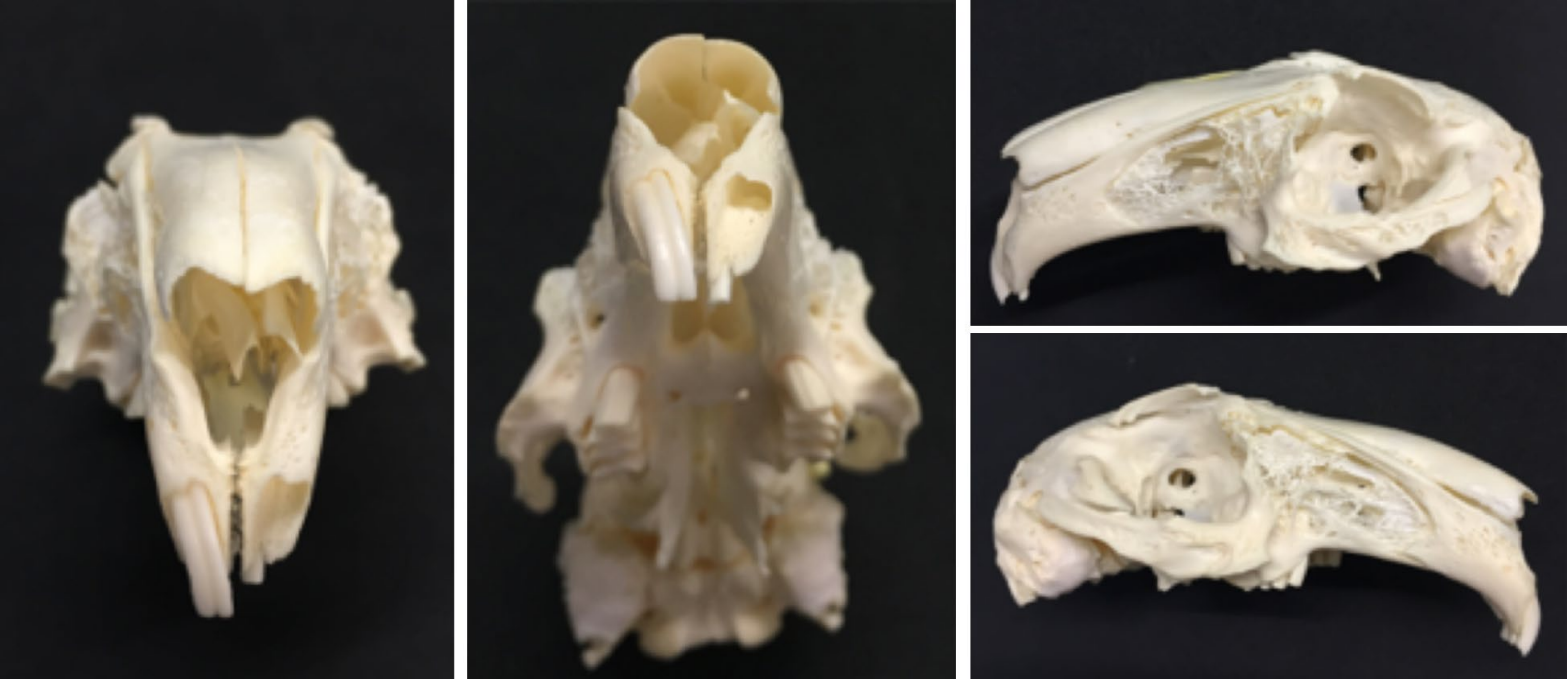
Osteological survey of New Zealand White rabbit skull. Preserved skull of a NZW rabbit (*Oryctolagus cuniculus*) showing the depth and orientation of the maxillary incisor alveolus after removing the central incisor. The alveolus penetrates into the maxillary bone laterally with strong curvature toward the palatal shelf. The tooth extends just shorts of the extensively aerated maxillary sinus

A skull of a NZW rabbit was obtained from the animal stock library at RWTH Aachen University Hospital (Germany) for inspection of the anatomical landmarks (Fig. 1).

### Microfocus computed tomography (Micro-CT) for imaging survey

**Fig. 2:**
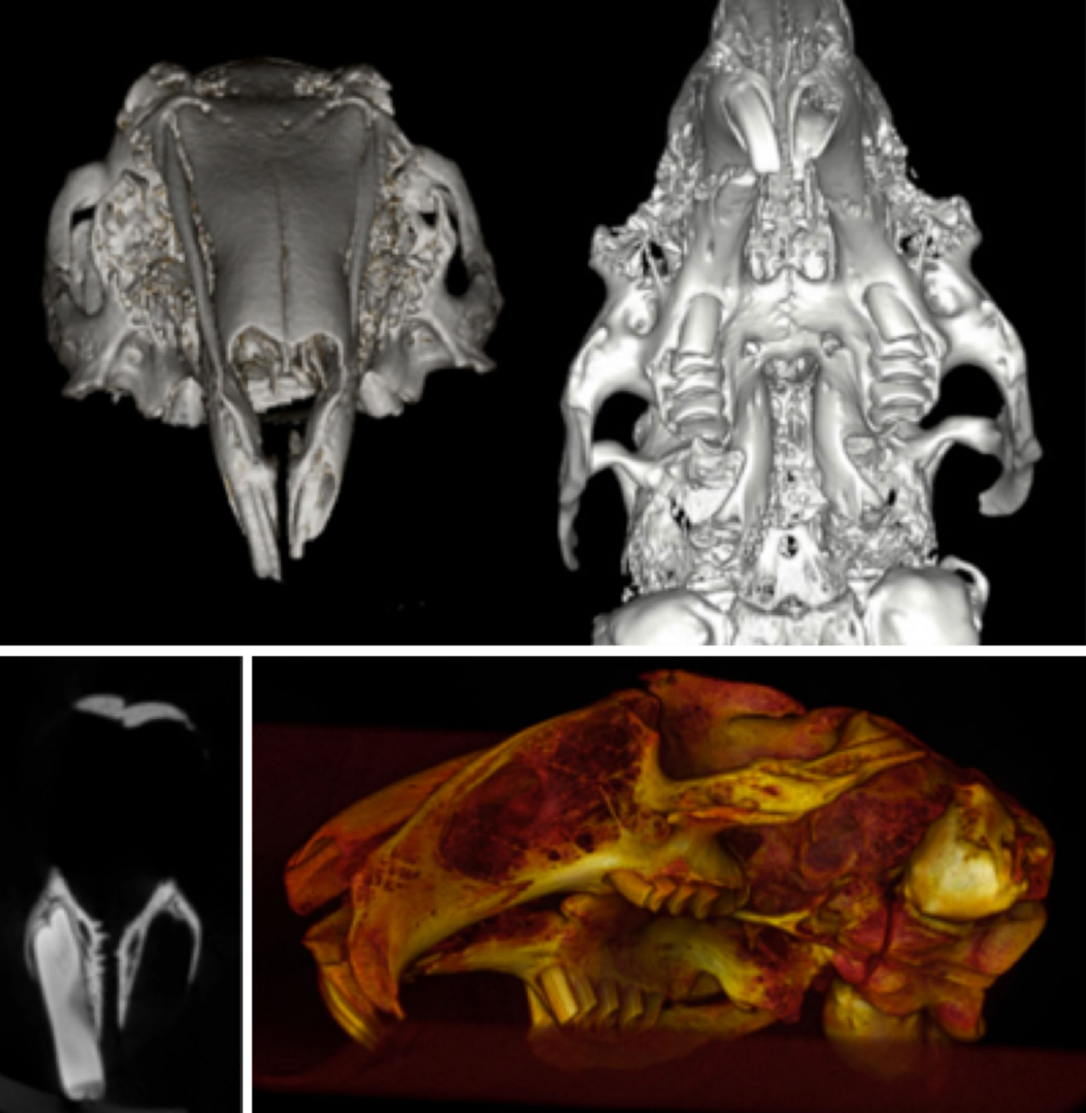
Imaging survey. Micro-CT three-dimensional reconstruction of a preserved skull of a NZW rabbit showing the depth and morphology of the extraction socket. Coronal sections at the level of the central incisors show thin bony plates separating the tooth from the nasal cavity

Imaging with Micro-CT of a rabbit skull were obtained of radiographic analysis of the skeletal anatomy for assessment of the feasibility of creating an alveolar cleft defect and planning of the cleft creation surgery. Images were evaluated using cross-sectional slices and rendered three-dimensional reconstruction (Fig. 2).

### Postmortem pilot alveolar cleft creation surgery and Micro-CT imaging

**Fig. 3:**
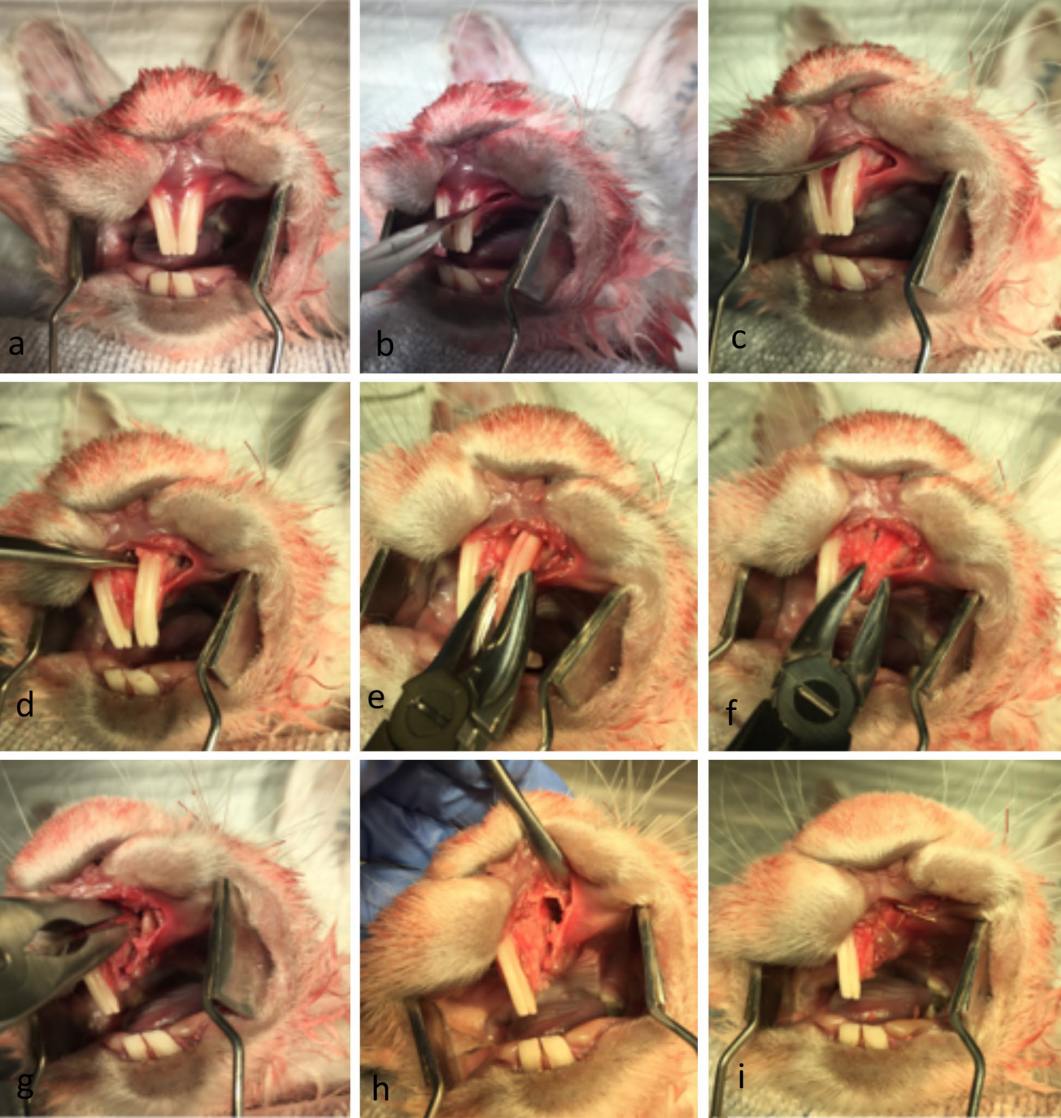
Postmortem pilot alveolar cleft creation surgery. **a**–**i** Postmortem pilot alveolar cleft creation surgery of a sacrificed NZW rabbit showing the modification of the extraction socket to expose the nasal lining. The defect can be completely covered by mucosa to allow healing and enable mucosal coverage of the cleft surfaces

**Fig. 4:**
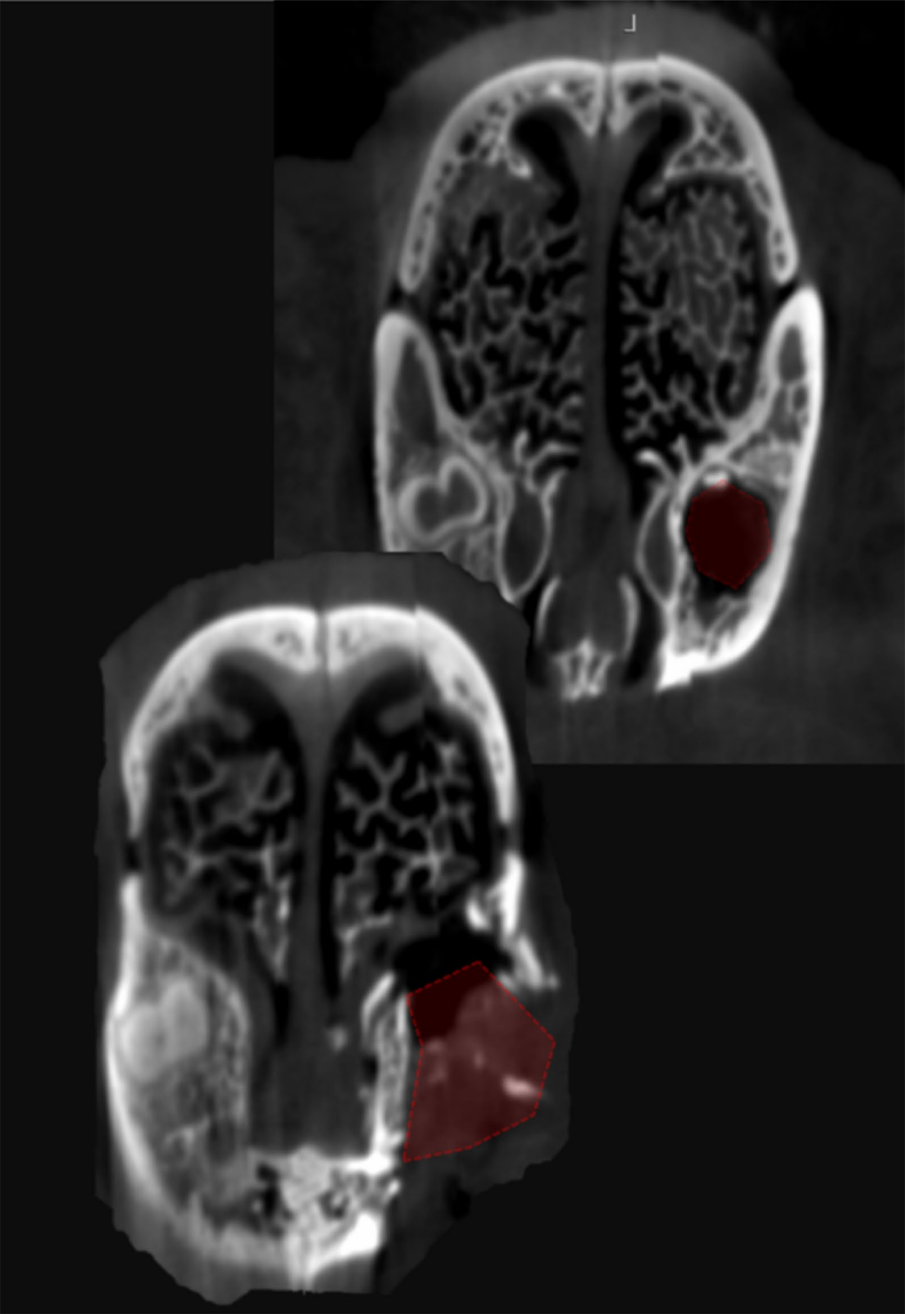
Postmortem imaging. Micro-CT imaging of the created cleft in a pilot postmortem surgery. Coronal view at the level of the anterior part of the extraction socket shows a complete cleft 8 mm wide extending from the oral cavity to the nasal cavity. Coronal view at the apical region of the extracted central incisor shows a residual defect with all bony walls intact

A pilot surgical operation of the planned procedure was conducted on a sacrificed rabbit head. There was no need for an animal ethical committee approval given that the procedure was performed on a sacrificed animal which was previously been used in another animal testing project. The cleft creation surgery was conducted according to the proposed procedure (Fig. 3). Post-operative microfocus computed tomography (Micro-CT) was obtained of the rabbit skull to evaluate the created postmortem defect (Fig. 4).

### In-vivo alveolar cleft creation surgery: animals, anesthesia, and housing

After evaluation of the correct position and volume of the cleft in the post-mortem pilot 16 8-week-old New Zealand White rabbits weighing 2.6–3.0 kg were operated in the same manner to prove the model in an in vivo setting. The procedure was conducted at the Animal Research Centre, Health Sciences Centre, Kuwait University. The project was subjected to strict animal testing protocol and the approval by the animal ethical committee at Animal Research Centre, Health Sciences Centre, Kuwait University. The rabbits were sedated 30 min prior to the experimental surgery with xylazine HCl 5 mg/kg by intramuscular injection and subsequently anesthetized by intravenous injection of 35 mg/kg of ketamine HCl. To ensure a high standard of animal care, a veterinarian was administering the sedation, anesthesia, and care-taking of the animals following an already used methodology [35, 36]. The animals were kept in separate cages and fed pellets and water throughout the duration of the study. The rabbits were cared of accordingly per protocol and observed by veterinarian until the end of the study.

### In-vivo alveolar cleft creation surgery in NZW rabbits (n = 16)

#### Preparation of surgical sites in maxillary alveolus

The surgery was performed with the rabbits in a supine position and under sterile conditions. A linear mucosal incision approximately was performed 2 cm lateral to the left central incisor and along the curvature of the tooth, extending to the distobuccal angle of the left central incisor, and then extended into the gingival margin of the left central incision on the facial aspect to the midline papilla. The gingiva and soft tissue were to expose the maxillary alveolus and the periodontal attachment of the central incisor tooth. Subsequently a flap was raised subperiosteally to expose the inferior nasal aperture. The nasal mucosa was freely protected and elevated using curved periosteal elevator taking care not to perforate the nasal mucosa. Lateral osteotomy along the lateral curvature of the left central incisor was performed using a rotary instrument with a round carbide bur to create a window exposing the root of the central incisor. The central incisor was then gently luxated toward the weakened lateral wall using a small dental elevator and eventually extracted using a veterinarian dental extraction forceps for rodents. Further osteotomy was carried to remove the superior and inferior bony plates with rongeur forceps to expose the nasal mucosa without injuring the mucosa. Bone wax then applied to the bony walls of the defect and the oral mucosa was approximated and sutured with five zeros resorbable Vicryl suture (Vicryl, Ethicon, USA) only on the medial and lateral sides. The central part of the wound was left open creating a pocket overlooking the bony defect. The defect was packed at the end with oxidised cellulose (Surgicel, USA). The nasal mucosa was left intact throughout the surgical procedure. The animals were allowed a period of 8 weeks for healing of the defect and the creation of maxillary alveolar defect.

The same surgical preparation and the strict protocol was carried at the second stage to expose the alveolar defect in preparation of bone grafting. A submarginal incision is made in the alveolar defect to separate the oral from the nasal mucosa. The bony defect was exposed and bony walls were lightly freshened with a round diamond bur. The rabbits were fed soft diet ad lib directly after the surgery. The rabbits were cared of accordingly per protocol and watched by veterinarian until the end of the study after 3 months.

### Post-operative cone beam computed tomography of the alveolar clefts

Postoperative imaging with Cone Beam CT of the rabbit skull was obtained 8 weeks after cleft creation surgery (Fig. 8).

## Results

### Osteological survey of New Zealand White rabbit skull (*O. cuniculus*)

A visual inspection of a skull of NZW rabbit revealed that in the area of interest to our study, the anterior maxilla of a NZW rabbit harboured two paired central incisors and two paired accessory palatal incisors. The central incisor was prominent and is in the form of half a circle and the accessory palatal incisor was smaller and roughly half the length of the central incisor. Examining a skull with an extracted central incisor revealed that the prominent tooth traversed the maxillary bone just below the nasal aperture leaving a thin layer of bone separating the alveolar socket from the nasal mucosa. The alveolar socket of an extracted central incisor formed a pocket-like cavity with a dimension of 7–8 mm made it an ideal model for alveolar cleft studies once the superior and inferior bony plate was resected to create a continuous defect simulating what is commonly seen in patients with congenital alveolar cleft (Fig. 1).

### Microfocus computed tomography (Micro-CT) for imaging survey

Three-dimensional Micro-CT reconstruction of the rabbit skull and the Micro-CT imaging revealed high level of accuracy and clear view of the maxillary alveolar socket for volume analysis and measurement of bony landmarks (Fig. 2).

### Postmortem pilot alveolar cleft creation surgery and Micro-CT imaging

Simulation of the alveolar cleft surgery on a sacrificed rabbit skull revealed the feasability of creating an adequately sized defect. The defect could be easily extended to the nasal mucosa to simulate a real clinical defect. Soft tissue closure and suturing of the mucosa was readily performed (Fig. 3). Three-dimensional reconstruction of the defect revealed a triangular alveolar cleft defect with a width of 8 mm that extended to the nasal cavity with the apex of the defect posting posteriorly and toward the depth of the alveolar socket. This simulates the clinical findings in patients with alveolar cleft defect (Fig. 4).

### In-vivo alveolar cleft creation surgery in NZW rabbits

**Fig. 5:**
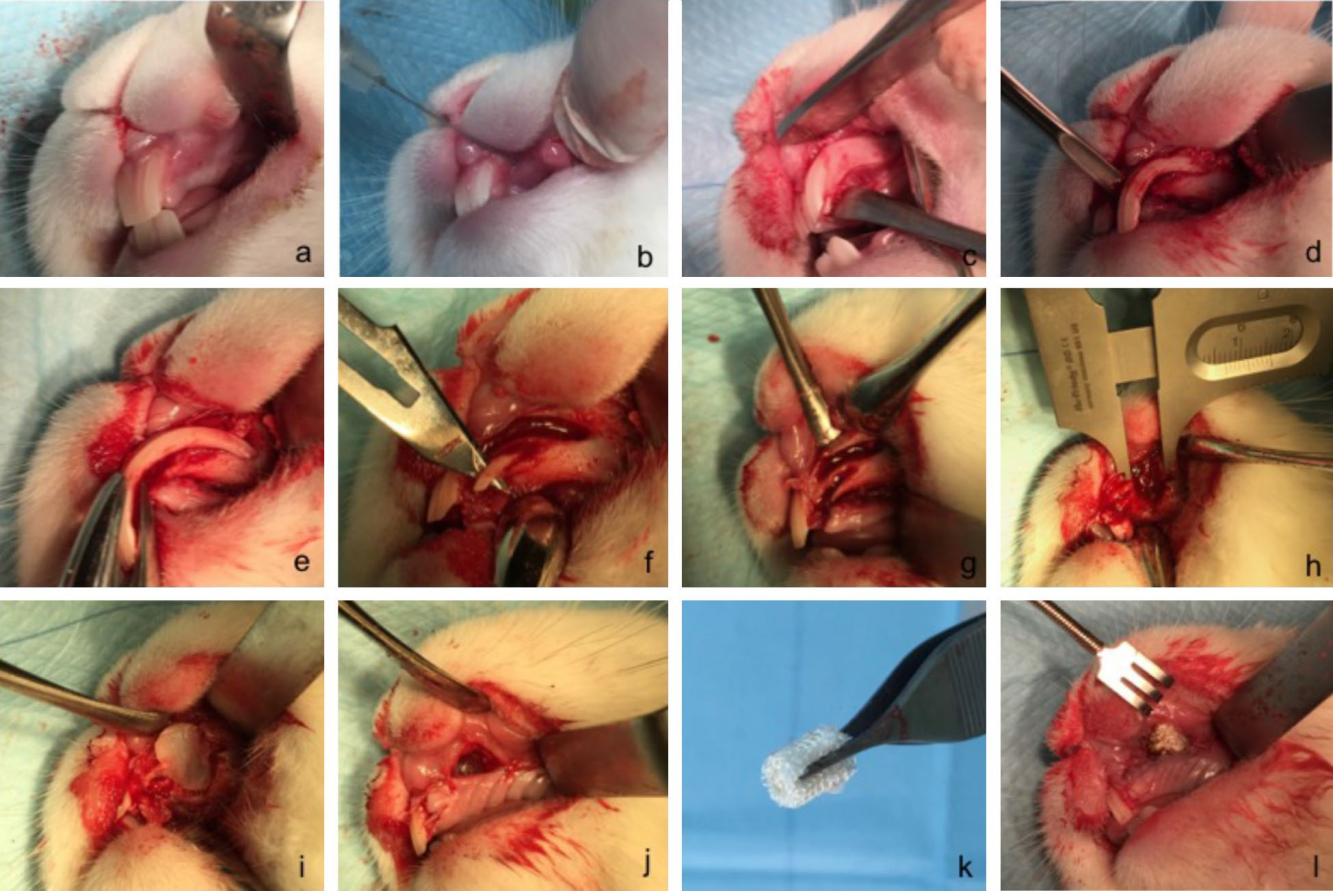
In-vivo alveolar cleft creation surgery. **a**–**l** In-vivo cleft creation surgery showing the modification of the extraction socket to expose the nasal lining. Distance material of bone wax and oxidized cellulose were used to keep the defect open and allow the mucosa to cover the cleft surfaces

**Fig. 6:**
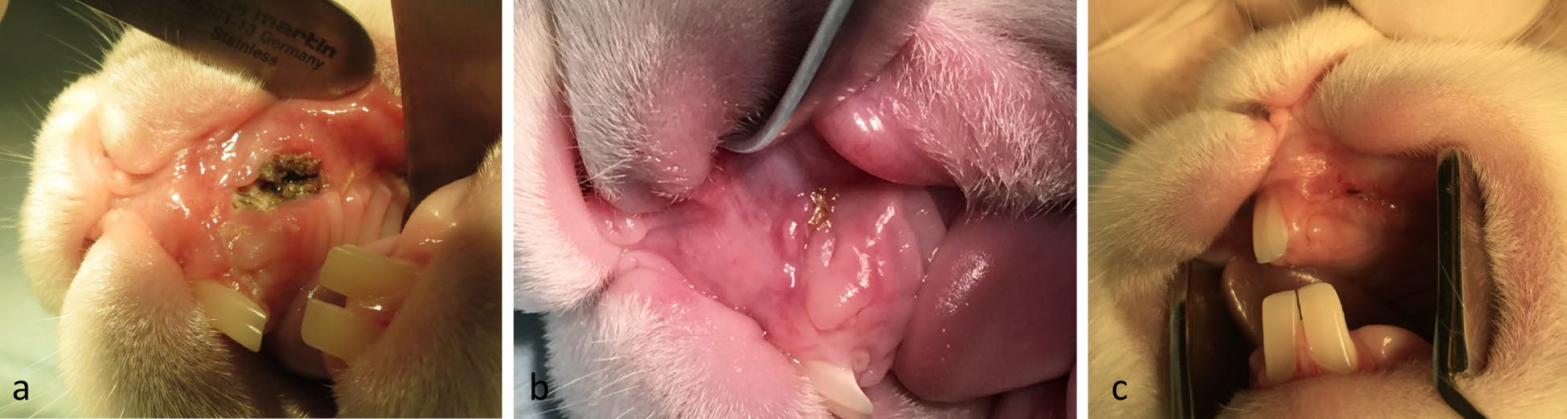
Wound healing. Healing of the surgery site 1 week after surgery (**a**) and 3 weeks after surgery (**b**). **c** Healed cleft site 8 weeks after surgery and before exposure of the alveolar cleft for bone grafting. Note the fistula overlying the cleft side indicating the establishment of an underlying bone defect

**Fig. 7:**
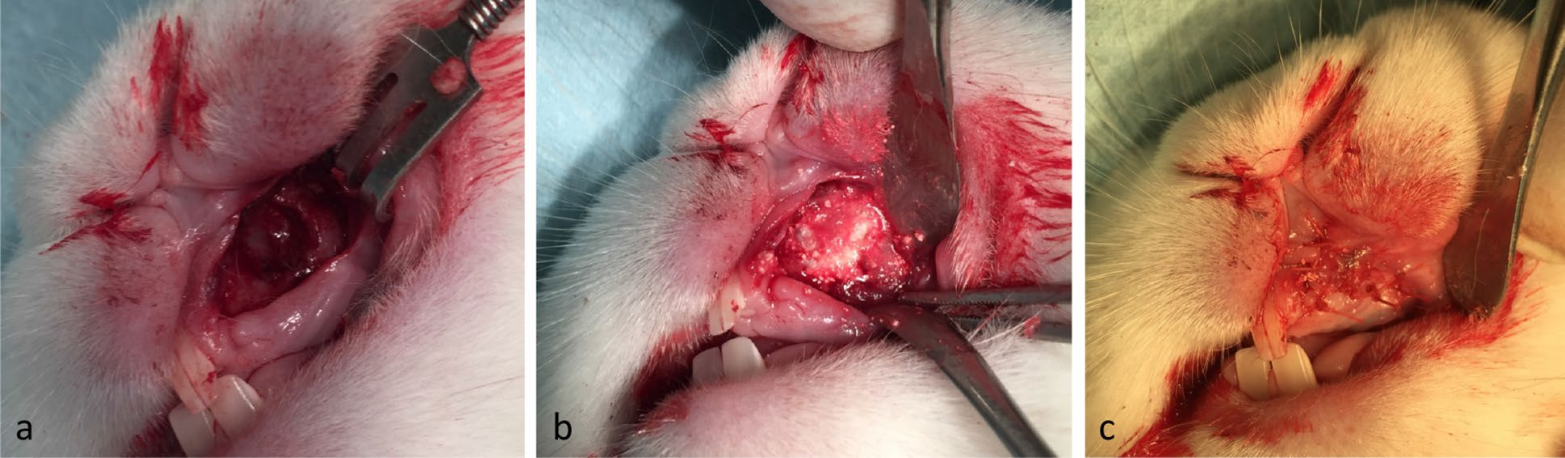
Grafting of the defect. **a** Healed alveolar cleft site 8 weeks after surgery and before grafting intervention. **b** Grafting intervention in the established alveolar cleft. **c** Final coverage of the cleft with oral mucosa. Complete coverage of the defect is achieved without wide mobilization of the soft tissue

The surgery for each rabbit took between 15 and 30 min, and all procedures could be carried out during Ketamine anesthesia without endotracheal intubation (Fig. 5). In a few cases an extra injection of Ketamine was required. Bleeding was minor and not a clinical issue during or after the cleft creation surgery. The animals were active and behaved adequately immediately after the surgery. They all started eating already during the first days after surgery. The animals were fed ad libitum well throughout the study. All the rabbits survived during the 8 weeks postoperatively until day of the sacrifice (Figs. 6, 7). The methodology and results of this in vivo-study will be reported more in detail in later separate papers.

### Post-operative computed tomography of the alveolar clefts

**Fig. 8:**
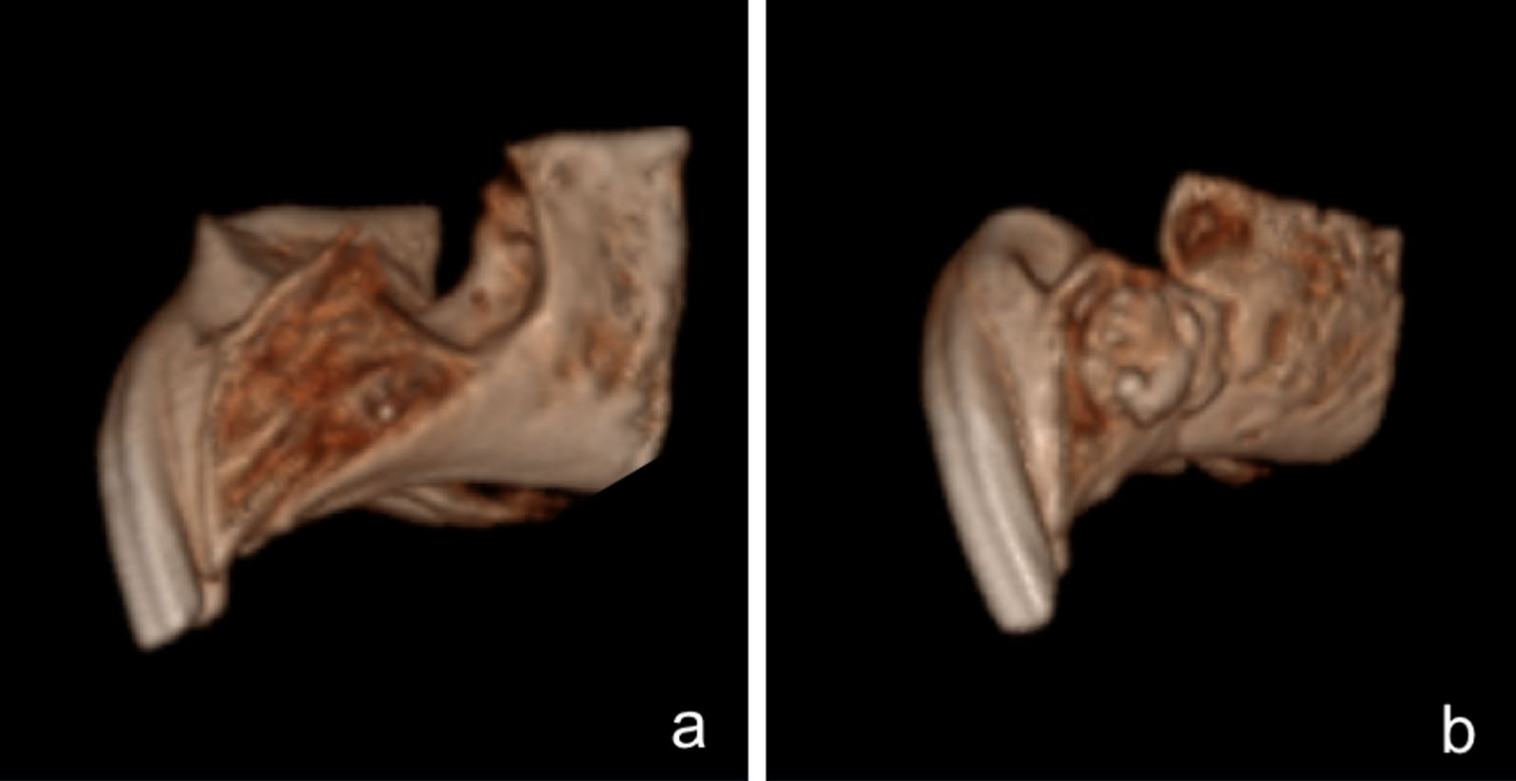
Postoperative imaging. **a** Cone Beam CT imaging of the created alveolar cleft after 8 weeks of healing shows a complete cleft extending from the oral cavity to the nasal cavity. **b** Alveolar cleft 8 weeks after grafting the defect with bone substitute material

After 8 weeks healing, computed tomography was carried out in one animal. A three-dimensional reconstruction of the defect revealed a triangular alveolar cleft defect with a width of 8 mm and extending to nasal cavity with the apex of the defect posting posteriorly and toward the depth of the alveolar socket was verified (Fig. 8).

## Discussion

Alveolar maxillary defects are unique defects with regard to their overlying soft tissues; the oral mucosa inside the mouth and the nasal mucosa as the nasal floor lining. The topography of such defect makes it susceptible to two biologically distinctive anatomical compartments each with a particular bio-environment. The aim of alveolar bone grafting is to obtain a proper reestablishment of these anatomical structures and provide a new bone structure that are acceptable in regard to volume and bone quality. Utilization of an animal model with a healed alveolar cleft defect, which mimics the three dimensional morphology in human patients with cleft lip and palate and extending to the nasal mucosa, would be best model to test the healing pattern of bone graft materials and to establish the proper anatomical structure.

Proper grafting of alveolar cleft deformities is an essential step to re-establish the dental arch in patients with cleft palate. This depends on the type of the grafting material that aims to restore form, volume and the functional establishment of a skeletal biological medium that would allow the eruption of the permanent teeth. Recent advances in tissue-engineered bone substitute materials and biomedical science have prompt further improvements in existing testing animal models to better evaluate the osteogenic efficacy and healing efficiency of the new grafting materials [4–8, 37].

Several models have been proposed as alveolar cleft model for testing of tissue-engineered bone replacement material. These ranging from mice, rats, rabbits, cats, dogs, swines, goats, sheep and monkeys [4, 9–24]. Prior description on rats models were able to create defects simulating alveolar defects because of their ease of handling and cost effectiveness, however, these defects tend to be significantly smaller in volume than human alveolar defects making it technically challenging to properly perform the grafting testing procedure [13, 17, 18, 21, 24].

The first description in the literature on an animal model for creating an alveolar cleft was reported by Harvold. He described the creation of an alveolar and palatal cleft 2 mm wide in two Rhesus monkeys [38]. The skeletal metabolism and bony macrostructure is small rodents tends to be far more active than bigger mammals and this may compromise the applicability of the animal testing findings. Thus bigger animals were considered a closer models to the human counterparts and able of mimicking skeletal defects and several attempts have been trying to describe further surgical techniques and modifications on previously reported methodologies mostly on monkeys and dog models [11, 12]. In recent years, attempts have been made to create more effective, economical and smaller animals to create acceptable alveolar cleft defects. Despite the increased descriptions of several animal models for alveolar bone grafting materials testing, most of the in vivo animal models using small animals were not able to function as reliable alveolar cleft model simulating that in human. This is mostly because of the limited anatomical size in these models which lead to difficulties in handling intraoral surgical procedures. This results in a compromise in designing and establishing proper sizable defects mimicking three-dimensional triangular defect extending to the nasal mucosa and the adjacent tooth as seen in human patients [12, 18, 39].

Most current reports in the literature illustrated the creation of simple non-anatomical defects in the maxilla of small animals by creating a maxillary or palatal window to establish a communication between the oral cavity and the nasal cavity, as described by Nguyen et al., Raposo-Amaral et al., Mostafa et al., Takano-Yamamoto et al. and Kim et al. in rats, and by Sawada et al. and Puumanen et al. in rabbits [17, 18, 21, 22, 39–41]. Xu et al. described the establishment of a cleft model in rats by extracting a molar tooth and applying bone wax [24]. Their model has succeeded in controlling the osseous healing process but the anatomical location of the defect does not correspond to a tridimensional maxillary alveolar cleft defects as encountered clinically. In some studies the defects were created and were not allowed to heal to establish a true non-healing critical defect and the insertion of the grafting material was performed simultaneously during the cleft creation surgery as seen in the models described by Takano-Yamamoto et al., Sawada et al., Puumanen et al., Pilanci et al. and Kim et al. [20, 22, 39–41]. The immediate grafting of the created defect leads to masking the effect of the native bone healing, and thus it would be difficult to attribute the new bone formation to the potential of the inserted graft rather than the native bone healing. El-Bokle et al. described a rabbit model for creating an alveolar cleft defect by extracting the central and lateral incisor and creating a wide defect and splitting the nasal mucosa to suture it the oral mucosa and leaving 1 cm defect [11]. In our opinion, a wide skeletal defect is plausible in creating alveolar cleft but 1 cm oronasal communication rarely correlates with clinical scenarios. A meta-analysis by Bykowski et al. evaluated the rate of oronasal fistula after primary cleft repair surgery and reported that most of the significant oro-nasal fistulas leading to clinical symptoms occurred posterior to the incisive foramen with the highest incidence at the soft-hard palate junction, and the lowest incidence of the fistula occurring at the maxillary alveolus [42]. In addition, splitting and suturing the nasal mucosa in a rabbit model is challenging given the anatomical limitation and would significantly increase the operating time and may compromise the health of the animal due to increased anesthetic requirement, compromise oral intake postoperatively, and increase the risk of aspiration and bleeding into the nasal cavity.

In our model we were able to achieve a reliable and consistent alveolar cleft in the maxillary front region and extending to the nasal mucosa, along with a nearby tooth which simulates the local anatomy of maxillary alveolar cleft in cleft lip and palate patients. Extracting an incisor by removing bone lateral to the incisor and luxate the tooth laterally has recently been described by Maslamani et al. in an experimental tooth replantation study [43]. Due to the extremely curved rabbit incisor it is not possible to extract through the longitudinal axis of the tooth but instead through lateral luxation after some bone removal.

Preparation of the cleft by keeping the nasal mucosa intact was possible and the surgery can be carried in 15–20 min and without general inhalation anesthesia or intubation, and without major risk of aspiration. The control of the space preventing it from bony ingrowth with simple bone wax seems to be consistently efficient in limiting bone healing, and the oxidized cellulose help in maintaining a spaced and aiding in prevent the collapse of the defect by extensive fibrous tissue. Allowing 8 weeks for cleft creation surgery seems to be enough to assume critically sized defect.

Our study shows that it is possible to produce a reliable and predictable animal model in rabbits to perform alveolar bone grafting. The surgical site area is of similar size and in the same region as that of pediatric population in human subjects, and the procedure does not require magnifying apparatus or micro instruments.

## Conclusions

A simple and predictable rabbit model with alveolar cleft for the clinical testing of e.g. tissue-engineering bone substitute materials can be created by following the existing anatomy and extraction of a central incisor tooth, modification of the extraction socket by extending it to the nasal mucosa, and the application of simple bone wax and oxidized cellulose material to help modulating the healing phase in the cleft area and avoid rapid bone generation and filling of the defect. Allowing 8 weeks of healing yields a predictable and good sized defect that can be used for later grafting procedures.

## Abbreviation

NZW: New Zealand White rabbit
